# Prenatal delta-9-tetrahydrocannabinol exposure alters fetal neurodevelopment in rhesus macaques

**DOI:** 10.1038/s41598-024-56386-7

**Published:** 2024-03-09

**Authors:** Kimberly S. Ryan, Joshua A. Karpf, Chi Ngai Chan, Olivia L. Hagen, Trevor J. McFarland, J. Wes Urian, Xiaojie Wang, Emily R. Boniface, Melanie H. Hakar, Jose Juanito D. Terrobias, Jason A. Graham, Scarlet Passmore, Kathleen A. Grant, Elinor L. Sullivan, Marjorie R. Grafe, Julie A. Saugstad, Christopher D. Kroenke, Jamie O. Lo

**Affiliations:** 1 Division of Maternal-Fetal Medicine, Department of Obstetrics and Gynecology, Oregon Health and Science University, 3181 SW Sam Jackson Park Road, Mail Code L458, Portland, OR 97239 USA; 2 Division of Neuroscience, Oregon National Primate Research Center, Oregon Health and Science University, Beaverton, OR USA; 3 Tissue Technologies Unit, Center for Virology and Vaccine Research, Beth Israel Deaconess Medical Center, Boston, MA USA; 4 Division of Reproductive and Developmental Sciences, Oregon National Primate Research Center, Oregon Health and Science University, Beaverton, OR USA; 5 Department of Anesthesiology and Perioperative Medicine, Oregon Health and Science University, Portland, OR USA; 6 Department of Pathology, Oregon Health and Science University, Portland, OR USA; 7 Integrated Pathology Core, Oregon National Primate Research Center, Oregon Health and Science University, Beaverton, OR USA; 8 Department of Psychiatry, Oregon Health and Science University, Portland, OR USA

**Keywords:** Cannabis, Delta-9-tetrahydrocannabinol, Neurodevelopment, Fetal Brain, Rhesus macaque, Pregnancy, Brain imaging, Intrauterine growth, Neurogenesis, Developmental neurogenesis, Translational research

## Abstract

Prenatal cannabis use is associated with adverse offspring neurodevelopmental outcomes, however the underlying mechanisms are relatively unknown. We sought to determine the impact of chronic delta-9-tetrahydrocannabinol (THC) exposure on fetal neurodevelopment in a rhesus macaque model using advanced imaging combined with molecular and tissue studies. Animals were divided into two groups, control (n = 5) and THC-exposed (n = 5), which received a daily THC edible pre-conception and throughout pregnancy. Fetal T2-weighted MRI was performed at gestational days 85 (G85), G110, G135 and G155 to assess volumetric brain development. At G155, animals underwent cesarean delivery with collection of fetal cerebrospinal fluid (CSF) for microRNA (miRNA) studies and fetal tissue for histologic analysis. THC exposure was associated with significant age by sex interactions in brain growth, and differences in fetal brain histology suggestive of brain dysregulation. Two extracellular vesicle associated-miRNAs were identified in THC-exposed fetal CSF; pathway analysis suggests that these miRNAs are associated with dysregulated axonal guidance and netrin signaling. This data is indicative of subtle molecular changes consistent with the observed histological data, suggesting a potential role for fetal miRNA regulation by THC. Further studies are needed to determine whether these adverse findings correlate with long-term offspring neurodevelopmental health.

## Introduction

Cannabis use, especially amongst reproductive aged individuals, has continued to rise due to changes in legalization trends and societal acceptability the last two decades^1^. In parallel, over the past decade prenatal cannabis use has doubled and cannabis is now the most commonly used illicit drug in pregnancy^1–5^. The American College of Obstetrics and Gynecology^2^ recommends that pregnant individuals, or those planning to become pregnant, should discontinue use of cannabis. However, cannabis is still frequently used to manage common pregnancy symptoms, such as nausea or sleep difficulties, for which alternative and safer treatment options are available^6,7^. Most concerning is that the main psychoactive ingredient in cannabis, delta-9-tetrahydrocannabinol (THC), readily crosses the placenta and cannabinoid receptor (CB1 and CB2) expression has been demonstrated within different regions of the fetal brain, including the prefrontal cortex^8–10^. Thus, there is concern for potential adverse fetal neurodevelopmental sequela following in utero cannabis exposure.

The existing literature is limited, but suggests that prenatal cannabis use is associated with an increased risk of stillbirth, small for gestational age infants, preterm delivery, and offspring neurodevelopmental morbidity, including autism spectrum disorder, attention deficit hyperactivity disorder and cognitive disabilities^11–13^. Babies prenatally exposed to cannabis display an exaggerated response to stimuli, sleep disruption, and a high-pitched cry, indicative of adverse neurological development^14^. Adolescents that were prenatally exposed to cannabis have been reported to experience increased inattention, hyperactivity, and impulsivity in addition to decreased school performance^15,16^. However, despite the significant public health relevance, there remains a critical gap in knowledge on the effects of prenatal cannabis exposure. At present there is insufficient evidence to establish a causal association between maternal cannabis use during pregnancy and offspring neurodevelopmental outcomes. Existing human studies are largely limited due to confounding variables including polysubstance use, sociodemographic factors, patient self-report, and retrospective observational data^17^.

The current paucity of knowledge is due in part to the lack of relevant preclinical models that have strong translation to human health. The rhesus macaque (*Macaca mulatta)* model is highly translational to humans and permits control of cofounding variables that limit human research. Specifically, the developmental trajectory of rhesus macaques across gestation, especially with respect to the development of the central nervous system, is similar to that of humans; rhesus macaque brains undergo similar phases of brain growth to humans throughout gestation^18^. Additionally, the rhesus macaque model allows for the precise control of timing, delivery, and dosing of cannabis exposure that recapitulates contemporary use.

To address this gap, we leveraged our translational rhesus macaque model of chronic THC edible consumption. With this model, we have previously demonstrated that in utero THC exposure significantly reduces placental perfusion and oxygenation^19^, and alters DNA methylation of key developmental genes in the placenta as well as multiple fetal tissues (e.g., lung, brain, and heart), including genes associated with autism spectrum disorder^20^. For this study, we extended our model to examine the impact of prenatal THC exposure on fetal brain function and development using a combination of advanced in utero imaging, molecular studies, and histologic assessment. Our group has experience studying the impact of maternal substance use on fetal brain development using in utero serial magnetic resonance imaging (MRI) across pregnancy^21^. Additionally, to facilitate the functional significance of these in utero anatomic findings on a molecular level, ex vivo studies were performed to further characterize cellular adaptations. Altered expression of microRNAs (miRNAs) and subsequent effects on post-transcriptional gene expression have been observed in neurodevelopmental disorders^22,23^, thus, examination of miRNA in fetal cerebrospinal fluid (CSF) was included in this study.

Our study focused on evaluating the potential harmful effects of chronic, prenatal THC exposure by studying its impact on fetal neurodevelopment in a rhesus macaque model using a multi-modal approach of advanced in utero imaging of the fetal brain across gestation combined with molecular and tissue studies.

## Results

### Fetal brain MRI

**Figure 1:**
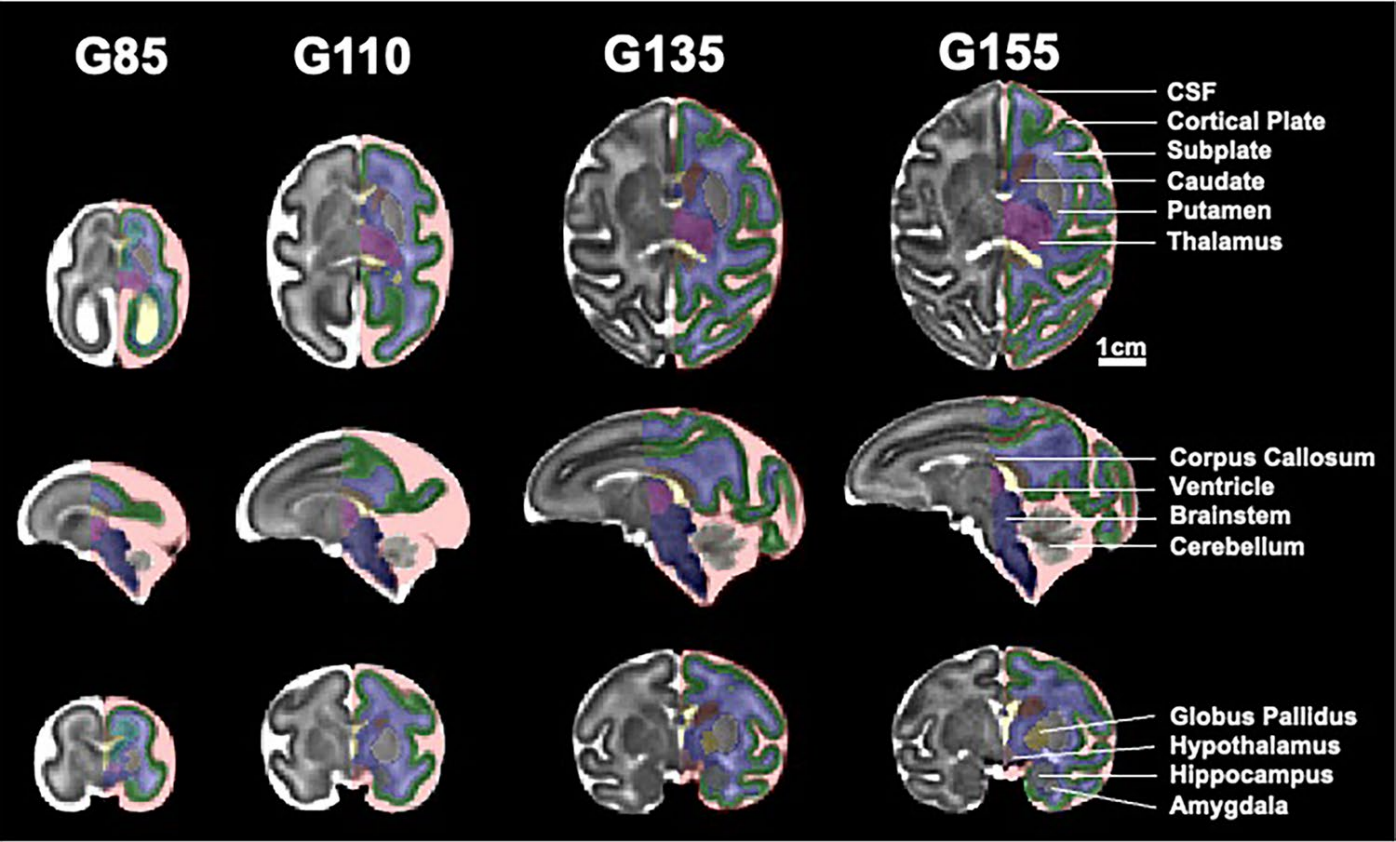
Study templates with regional segmentation overlays. Representative axial (top row), sagittal (middle row), and coronal (bottom row) views of the four T_2_-weighted gestational age study templates with color-coded segmentations partially overlaid. Regions corresponding to external CSF (red), cortical plate (green), fetal white matter (blue), germinal matrix (cyan), ventricles (yellow), corpus callosum (bronze), thalamus (magenta), hypothalamus (puce), caudate (brown), putamen (silver), globus pallidus (gold), hippocampus (grey), amygdala (light grey), cerebellum (tan), and brainstem (indigo) are visible. Scale bar represents 1 cm.

**Figure 2:**
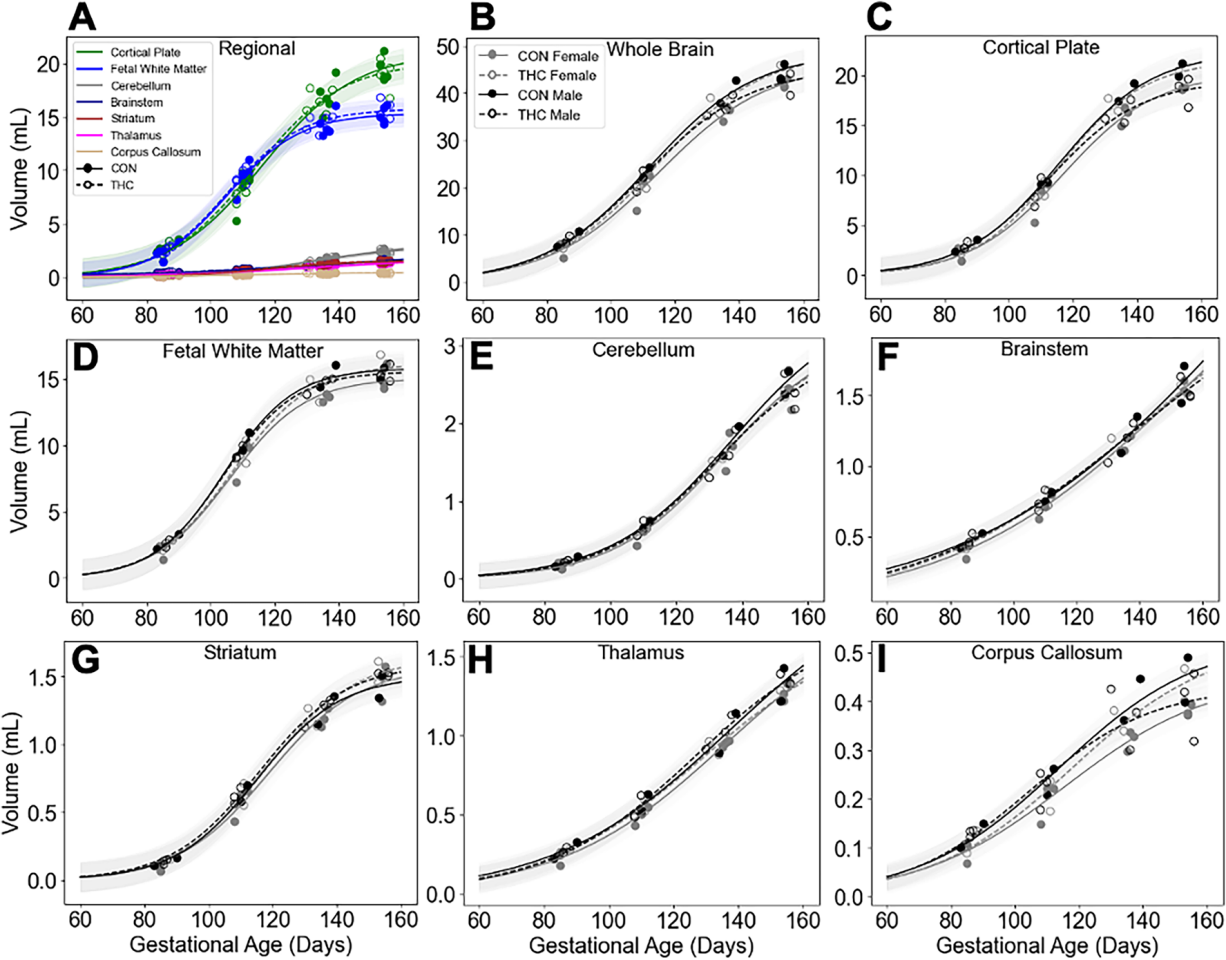
Comparisons of volumetric regional brain development did not demonstrate significant effects of treatment or sex. Individual regional brain volumes plotted by treatment only (**A**) and sex and treatment (**B**–**I**) with sigmoidal curve fits to guide the eye (shading indicates 95% CI). Brain regions identifiable at each timepoint are plotted as solid points for controls, and open points for THC-exposed animals. Curve fits with solid representing control, and dashes representing treatment are shown with for regions color coded to the corresponding segmentation map (**A**). Individual whole brain (**B**), cortical plate (**C**), fetal white matter (**D**), cerebellum (**E**), brainstem (**F**), thalamus (**G**), corpus callosum (**H**), and striatum (**I**) volumes are plotted for control (solid points and lines) and THC exposed (open points and dashes) for animals. Sex is indicated by color; grey for females, and black for males. While whole brain and regional volumes increased significantly with age (LME, all *p* < 0.0001) volumes did not differ significantly by treatment or sex (**B**–**I**) (all *p* > 0.05). However, a significant 3-way interaction between age, sex, and treatment was identified in the cortical plate (C; β =  − 67.3, SE = 31.7, *p* = 0.044, CI 95% [− 126.9, − 7.5]) and corpus callosum (I; β =  − 1.8, SE = 0.72, *p* = 0.021, [− 3.1, − 0.42]) described by a pattern of increased volume for THC-exposed females relative to controls, and decreased volume in THC-exposed males relative to controls.

In utero MRI was used to assess longitudinal changes in neurodevelopment throughout gestation at four timepoints: gestational day (G)85, G110, G135, and G155 (Fig. 1). Mixed effect model analysis revealed that whole brain and regional volumes (Fig. 2) increased significantly with age across G85, G110, G135, and G155 (all *p* < 0.0001) in both THC-exposed and control fetuses. While we did not observe a main effect of treatment or sex, our analysis revealed a significant interaction between gestational age, sex, and treatment for volume of the cortical plate (β =  − 67.3, SE = 31.7, *p* = 0.044, CI 95% [− 126.9, − 7.5]) and corpus callosum (β =  − 1.8, SE = 0.72, *p* = 0.021, [− 3.1, − 0.42]) (Table 1). THC-exposed females exhibited larger cortical plate volumes than control females, while THC-exposed males displayed smaller cortical volumes than control males on average across all four gestational timepoints. This pattern of reduced male, and increased female growth, following THC exposure is also reflected in the corpus callosum, with the exception of the G85 timepoint, in which THC-exposed males exhibited larger volumes than controls (Fig. 2). Although consistent between regions, these subtle differences in volumetric growth between males and females should be interpreted in the context of the small sample size, and comparisons across multiple brain regions.

**Table 1:** Mean volumes (mL) by gestational age, treatment, and sex.

Age	G85				G110				G135				G155			
Treatment	Control		THC		Control		THC		Control		THC		Control		THC	
Sex	M	F	M	F	M	F	M	F	M	F	M	F	M	F	M	F
n	2	3	3	2	2	3	3	2	2	3	3	2	2	3	3	2
Cortical plate* *mean (SD)*	2.98 (0.83)	2.27 (0.69)	2.91 (0.38)	2.62 (0.67)	9.21 (0.17)	7.63 (2.04)	8.15 (1.48)	8.66 (1.02)	18.31 (1.27)	16.01 (0.94)	16.14 (1.23)	17.06 (0.89)	20.55 (0.89)	18.70 (0.11)	18.44 (1.49)	20.17 (0.43)
Fetal white matter	2.77 (0.80)	2.09 (0.57)	2.62 (0.28)	2.47 (0.55)	10.32 (0.97)	8.94 (1.45)	9.39 (0.54)	9.52 (1.20)	15.23 (1.16)	13.64 (0.30)	14.58 (0.65)	14.11 (1.20)	15.44 (0.63)	15.03 (0.99)	15.41 (0.66)	16.00 (1.18)
Corpus callosum*	0.13 (0.03)	0.09 (0.02)	0.13 (0.01)	0.11 (0.03)	0.24 (0.04)	0.20 (0.04)	0.22 (0.04)	0.21 (0.04)	0.41 (0.06)	0.32 (0.02)	0.37 (0.06)	0.36 (0.03)	0.44 (0.07)	0.38 (0.01)	0.40 (0.07)	0.43 (0.05)
Ventricles	0.72 (0.12)	0.61 (0.13)	0.66 (0.03)	0.67 (0.07)	0.30 (0.03)	0.26 (0.03)	0.29 (0.05)	0.26 (0.05)	0.59 (0.10)	0.51 (0.04)	0.49 (0.06)	0.53 (0.08)	0.65 (0.12)	0.58 (0.02)	0.56 (0.07)	0.64 (0.11)
Germinal matrix	1.38 (0.32)	1.10 (0.21)	1.35 (0.11)	1.32 (0.20)												
Brainstem	0.47 (0.07)	0.40 (0.06)	0.47 (0.04)	0.46 (0.06)	0.78 (0.04)	0.71 (0.09)	0.75 (0.07)	0.77 (0.07)	1.23 (0.18)	1.18 (0.06)	1.18 (0.14)	1.15 (0.06)	1.58 (0.19)	1.56 (0.04)	1.54 (0.08)	1.53 (0.06)
Cerebellum	0.23 (0.09)	0.19 (0.05)	0.22 (0.02)	0.20 (0.03)	0.70 (0.06)	0.60 (0.16)	0.63 (0.11)	0.66 (0.01)	1.78 (0.27)	1.66 (0.25)	1.60 (0.31)	1.53 (0.02)	2.52 (0.20)	2.44 (0.26)	2.40 (0.23)	2.38 (0.06)
Thalamus	0.28 (0.07)	0.23 (0.04)	0.27 (0.02)	0.26 (0.03)	0.58 (0.08)	0.50 (0.06)	0.53 (0.08)	0.56 (0.05)	1.02 (0.18)	0.95 (0.02)	1.02 (0.11)	0.92 (0.06)	1.33 (0.15)	1.27 (0.05)	1.35 (0.04)	1.25 (0.05)
Hypothalamus					0.10 (0.01)	0.09 (0.01)	0.10 (0.01)	0.10 (0)	0.11 (0.01)	0.11 (0.01)	0.12 (0.01)	0.12 (0.01)	0.13 (0.01)	0.13 (0.01)	0.14 (0.01)	0.14 (0.01)
Hippocampus					0.24 (0.02)	0.20 (0.02)	0.21 (0.02)	0.23 (0.02)	0.27 (0.02)	0.25 (0.01)	0.26 (0.03)	0.26 (0)	0.32 (0.01)	0.29 (0.02)	0.32 (0.01)	0.32 (0.02)
Amygdala					0.09 (0.01)	0.08 (0.01)	0.09 (0.01)	0.09 (0.01)	0.12 (0.01)	0.11 (0.01)	0.12 (0.01)	0.12 (0.02)	0.16 (0)	0.15 (0)	0.16 (0.01)	0.16 (0.02)
Striatum	0.14 (0.04)	0.10 (0.03)	0.14 (0.02)	0.14 (0.03)	0.64 (0.09)	0.57 (0.12)	0.62 (0.06)	0.63 (0.11)	1.25 (0.14)	1.19 (0.07)	1.25 (0.11)	1.20 (0.09)	1.43 (0.12)	1.46 (0.13)	1.52 (0.01)	1.53 (0.11)
Caudate					0.15 (0.02)	0.12 (0.01)	0.13 (0.01)	0.14 (0.03)	0.46 (0.07)	0.44 (0.04)	0.43 (0.02)	0.44 (0.04)	0.51 (0.05)	0.53 (0.05)	0.52 (0.03)	0.57 (0.06)
Putamen					0.49 (0.06)	0.45 (0.11)	0.49 (0.06)	0.50 (0.09)	0.61 (0.04)	0.58 (0.02)	0.62 (0.06)	0.58 (0.04)	0.65 (0.04)	0.69 (0.08)	0.73 (0.04)	0.71 (0.04)
Globus pallidus									0.19 (0.03)	0.18 (0.01)	0.20 (0.03)	0.18 (0.01)	0.27 (0.03)	0.25 (0.01)	0.26 (0.01)	0.25 (0.01)
Whole Brain	9.11 (2.38)	7.08 (1.78)	8.77 (0.87)	8.26 (1.66)	23.20 (1.50)	19.79 (4.01)	20.97 (2.37)	21.69 (2.61)	40.31 (3.39)	35.92 (1.67)	37.13 (2.25)	37.37 (2.41)	44.55 (2.36)	42.00 (0.96)	42.24 (2.41)	44.56 (1.97)

Regional and whole brain mean volumes in mL (standard deviations in parentheses) for animal groups by age, treatment, and sex. Missing cells denote ages where anatomy was not present or unable to be resolved. Standard deviation of (0) represents values less than 0.01. *p < 0.05 for age*treatment*sex interaction; linear mixed effects model.

### Fetal pathology

Fetal tissue was collected by necropsy at G155 (equivalent to the late third trimester in human pregnancies) following cesarean delivery. Gestational length and fetal weights were similar between control and THC-exposed fetuses. Gross and histopathological assessments were performed by trained veterinary pathologists on representative tissues (Supplemental Table S1). As previously reported^19^, THC-exposed male fetuses (n = 3) had smaller testes than the control male fetuses (n = 2). In addition, the adrenal glands of 3 of the THC-exposed fetuses were larger (0.34–0.41 g) than control fetuses (0.21–0.3 g), but this was not significant (*p* = 0.598). Otherwise, gross examination of representative tissues did not have significant findings between treatment groups.

### Fetal brain histology

**Figure 3:**
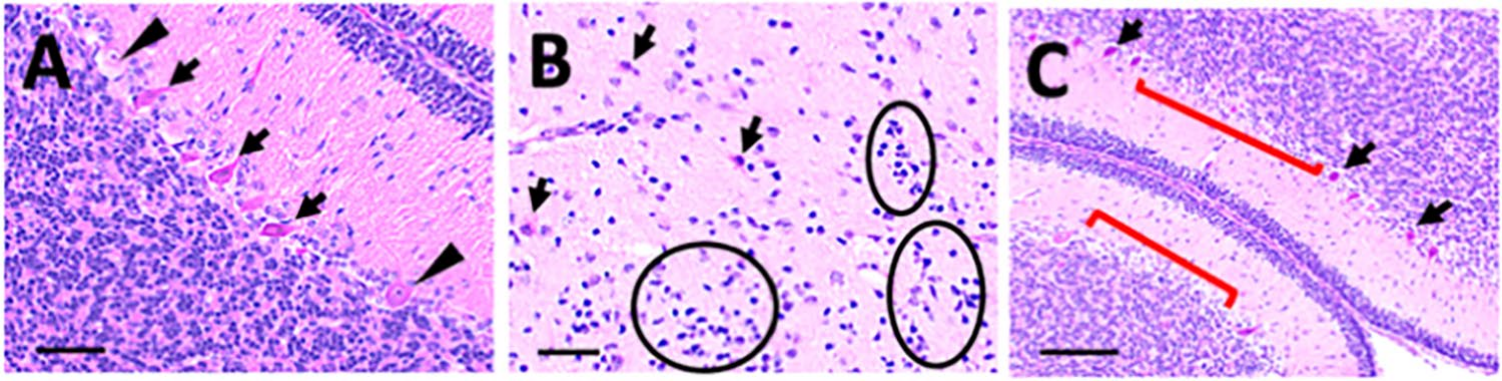
Prenatal THC exposure results in ischemic histopathologic changes in the fetal brain in a rhesus macaque model. Representative images of H&E stained fetal brain sections. (**A**) Cerebellum with acute ischemic changes demonstrated by shrunken, hypereosinophilic Purkinje cells with dark, shrunken nuclei (arrows). Intact Purkinje cells have lighter, less pink cytoplasm and large nuclei with prominent nucleoli (arrowheads).Magnification bar = 50 μm. (**B**) Parietal white matter with reactive astrocytes and microglial activation, Magnification bar = 50 μm.Representative reactive astrocytes demonstrate prominent cytoplasm and fibrillary processes (arrows). Representative microglial clusters consist of groups of haphazardly arranged small, elongated, angular to irregularly shaped nuclei (encircled). (**C**) Cerebellum with acute Purkinje cell death (arrows) and focal Purkinje cell dropout (red bracket), indicating earlier cell death, Magnification bar = 100 μm.

Histologic section of multiple fetal brain regions were examined, including prefrontal cortex, occipital cortex, basal ganglia, thalamus, corpus callosum, hippocampus, brain stem and cerebellum. Three sections per each brain region were evaluated. Histological examination of the fetal brains by trained neuropathologists (M.H.H. and M.R.G.) blinded to treatment groups revealed that all THC-exposed fetal brains (n = 5) demonstrated acute ischemic changes, especially in the cerebellum (Fig. 3A), and findings suggestive of some degree of ischemic white matter injury, including reactive astrocytosis (Fig. 3B) and increased microglial clusters (Fig. 3C) particularly within the temporal and parietal lobes. In addition, within the cerebellum, focal Purkinje cell dropout, indicating earlier Purkinje cell death and longer term injury, were observed (Fig. 3C). None of these histological findings were observed in control fetal brains. Both neuropathologists were in agreement regarding all histological findings. Brain weights were not significantly different between treatment groups (Supplemental Table S1).

#### Multiplex immunohistochemistry

**Figure 4:**
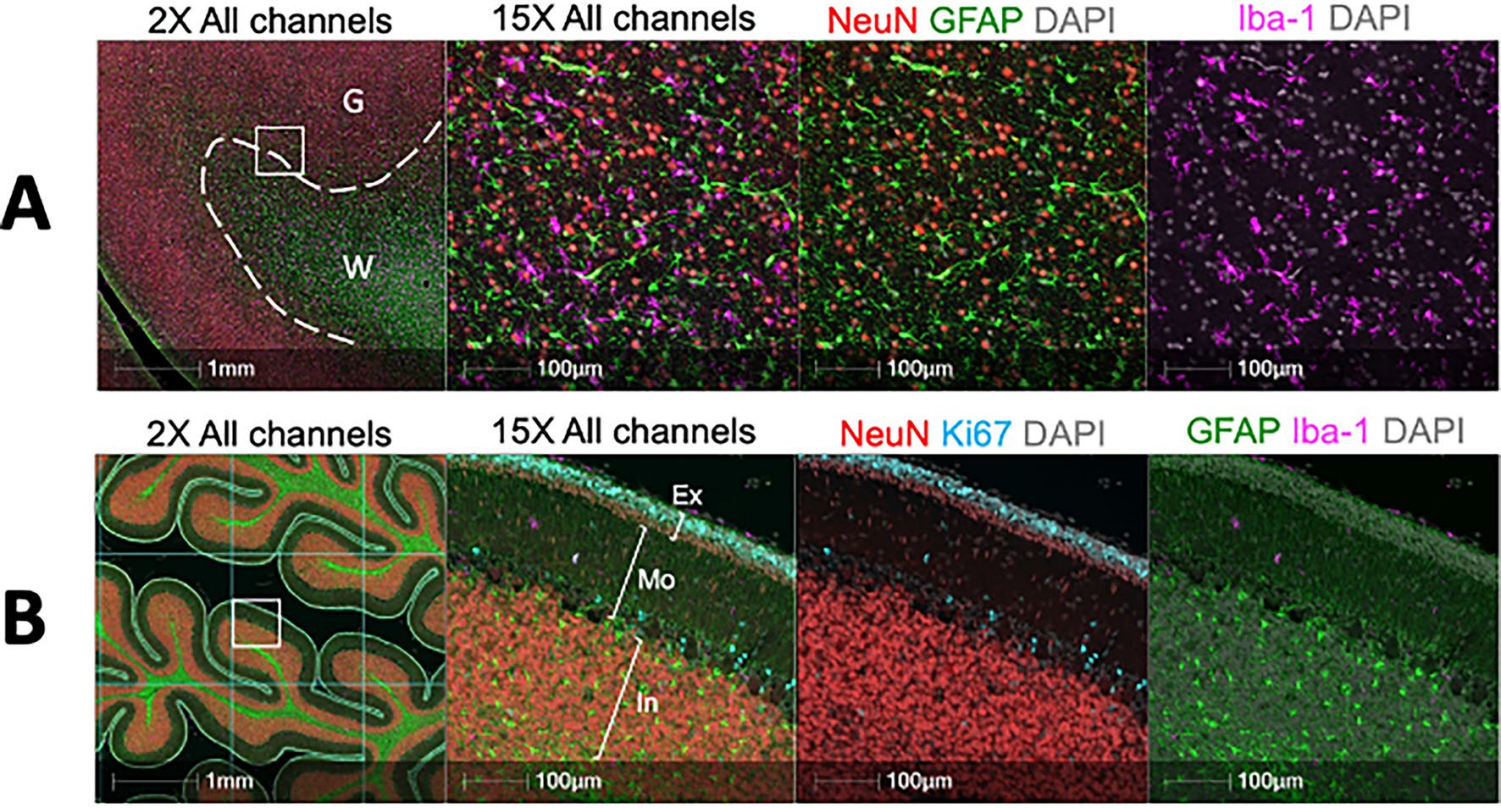
**A** Multiplex immunofluorescence of the fetal prefrontal cortex. FFPE sections were stained for neurons (NeuN—Red), microglia (Iba-1—Magenta) and astrocytes (GFAP—Green) by multiplex immunofluorescence. Dotted line in 2× panel represents the boundary between grey matter (G) and white matter (W) as differentiated by NEuN and GFAP staining. **B** Muliplex immunofluorescence image of the fetal cerebellum. FFPE sections were stained for neurons (NeuN—Red). Microglia (Iba-1—Magenta), Ki67 (Cyan) and astrocytes (GFAP—Green) by multiplex immunofluorescence. The external granule layer (Ex), Molecular layer (Mo) and internal granule layer (In) are highlighted in the 15× panel. For quantification, the tissue was divided by a grid and annotations representations representing each layer were drawn in 10 randomly selected grid squares per cerebellum.

To further assess the effect of prenatal THC exposure on the developing fetal brain, tissues were stained by multiplex immunofluorescence for the microglial marker, Iba-1, the neuronal marker, NeuN, the radial glia and astrocyte marker, GFAP, (Fig. 4A) and the proliferative marker, Ki-67 (Supplemental Fig. S1). A significant increase in Iba-1 area staining in the prefrontal cortex white matter (4.0% $\pm$ 2.0 vs. 1.9% $\pm$ 1.3 of the total annotated white matter area, *p* = 0.007) and a non-significant trend in the prefrontal cortex grey matter (2.5% $\pm$ 1.3 vs. 1.3% $\pm$ 1.0 of the total annotated grey matter area, *p* = 0.07) was observed in THC-exposed versus control. Similarly, there was a non-significant trend of increased GFAP area staining in the prefrontal cortex grey matter (30.7% $\pm$ 8.5 vs. 22.8% $\pm$ 11.1 of the total annotated grey matter area, *p* = 0.14) and white matter (51.4% $\pm$ 9.6 vs. 43.7% $\pm$ 12.6 of the total annotated white matter area, *p* = 0.16) in THC-exposed versus control. Prefrontal cortex grey matter showed similar frequency of Ki67+ cells (1.3% $\pm$ 0.3 vs. 1.32% $\pm$ 0.2, *p* = 0.36) and NeuN+ cells (55.8% $\pm$ 6.1 vs. 53.2% $\pm$ 6.2, *p* = 0.68) in THC-exposed versus control. Prefrontal cortex white matter multiplex immunofluorescence also showed similar frequency of Ki67+ cells (2.4% $\pm$ 0.5 vs. 2.2% $\pm$ 0.5, *p* = 0.14) and NeuN + cells (35.9% $\pm$ 10.0 vs. 34.4% $\pm$ 9.0, *p* = 0.90).

In the developing fetal brain, cerebellar granule neurons, the biggest neuronal population of the human nervous system^24,25^, are derived from precursors in the external granule cell layer^26^. In agreement with this, multiplex immunofluorescence staining of fetal cerebellum sections clearly showed this layer of Ki67+ cells on the surface of the cerebellum (Fig. 4B). Quantification of the frequency of Ki67+ cells in the external granule layer demonstrated similar levels of proliferating cells (62.7% $\pm$ 0.4 vs. 66.6% $\pm$ 0.6, *p* = 0.43) between THC-exposed and control animals. The expression of Iba-1 and GFAP was also quantified in the external granule layer, the molecular layer and the internal granule layer of the cerebellum. There was no significant difference in Iba-1 staining area in the cerebellum external granule layer (0.31% $\pm$ 0.15 vs. 0.16% $\pm$ 0.11, *p* = 0.29), molecular layer (1.39% $\pm$ 0.80 vs. 0.86% $\pm$ 0.49, *p* = 0.56), and internal granule layer (1.21% $\pm$ 0.99 vs. 0.61% $\pm$ 0.41, *p* = 0.56) of THC-exposed compared to control fetuses. In contrast, there was a non-significant trend of decreased GFAP staining﻿ area observed in the cerebellum external granule layer (27.4% $\pm$ 11.5 vs. 41.4% $\pm$ 5.36, *p* = 0.06), molecular layer (30.1% $\pm$ 7.10 vs. 35.1% $\pm$ 9.92, *p* = 0.41), and internal granule layer (30.2% $\pm$ 6.21 vs. 35.3% $\pm$ 7.82, *p* = 0.46) of THC-exposed compared to control animals.

### Fetal cerebrospinal fluid (CSF) miRNA qPCR

**Figure 5:**
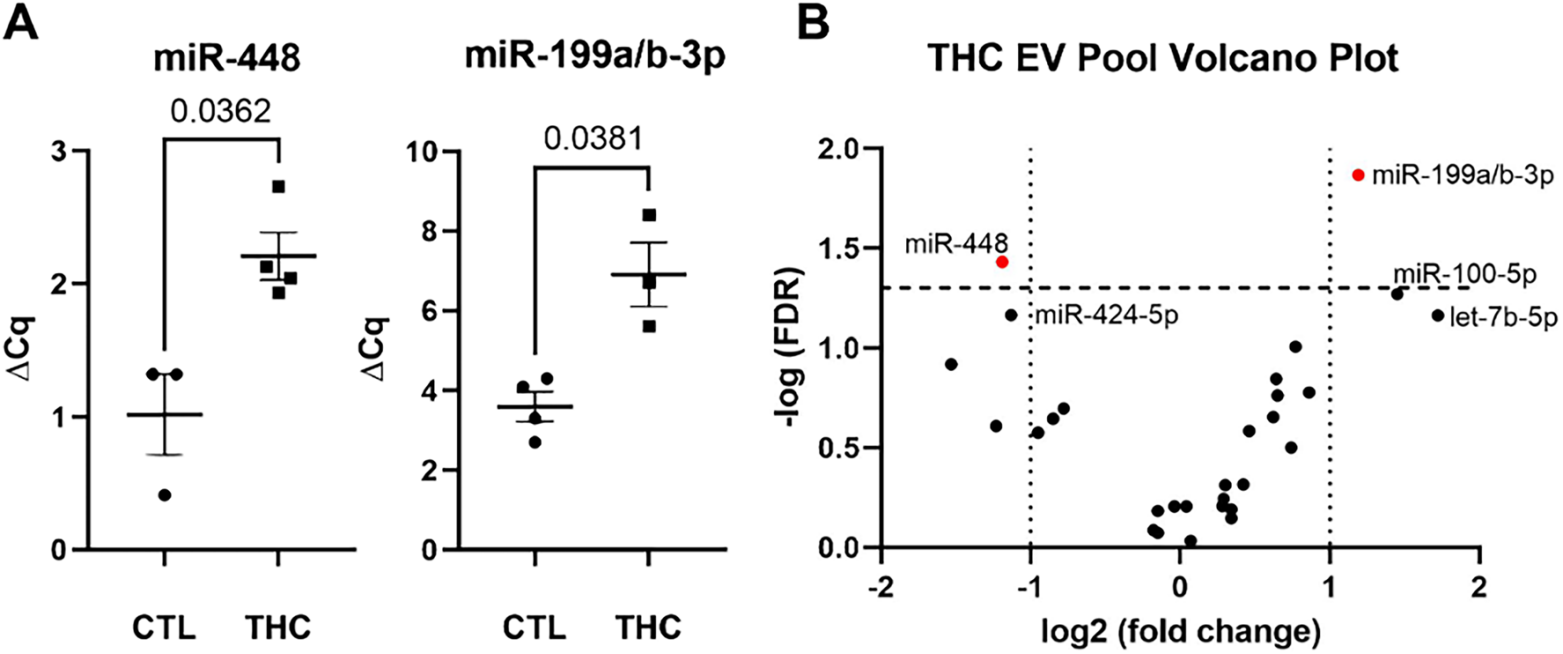
In utero THC exposure alters miRNA expression in fetal cerebral spinal fluid. (**A**) The qPCR data analysis revealed a significant (*p* < 0.05) difference in levels of miR-448 and miR-199a/b-3p in the THC-exposed (THC) versus the control (CTL) fetal cerebral spinal fluid. (**B**) The volcano plot shows a greater than twofold change in miR-448 and miR-199a/b-3p in THC-exposed versus control fetal cerebral spinal.

To better understand the potential underlying pathways associated with the fetal neurodevelopmental effects of prenatal THC exposure, miRNA analysis was performed on fetal CSF that was processed by size exclusion chromatography (SEC) to enrich for extracellular vesicles (EVs). An equal volume of the CSF EV fractions was used as input for the qPCR assays, as the amount of RNA in these sample is too small to measure by any method in order to determine concentration. Our previous publications support that pooled fractions 6–9 consistently contain protein markers for EVs in human CSF^27^ (Figs. 3, 4, 5) and plasma (Fig. 2).^28^ The miRNA qPCR data from the putative CSF EVs (pooled fractions 6–9) revealed a significant p-value (*p* < 0.05) with greater than twofold reduction in miR-448 (*p* = 0.036) and a greater than twofold increase in miR-199a/b-3p (*p* = 0.038) (Fig. 5) in THC-exposed versus control fetal CSF.

The study did not include an inter-assay control such as an exogeneous miRNA spike-in but previous studies utilizing CSF and/or CSF enriched EVs, demonstrated no evidence of qPCR inhibition. This study aligns with previous studies using human CSF enriched EVs, where we typically see ~ 60–120 miRNAs being amplified. For this data set, the average number of miRNAs for the control group was 77 and for the THC treated group the miR number was 86 and there was no significant difference between the number of amplifications that passed the qPCR performance cut-offs (*p* = 0.70). Additionally, the mean Cq for the THC treated group was 30.6 and the control group was 31 (Supplemental Fig. S2).

Predicted mRNA targets of the 2 THC-regulated miRNAs were assessed by ingenuity pathway analysis (IPA). IPA indicates that the mRNAs potentially targeted by these 2 miRNAs are in canonical pathways associated with axonal guidance, netrin signaling, and cardiac hypertrophy. In addition, IPA also implicated miR-448 and miR-199a/b-3 in developmental disorders and neurological and reproductive system diseases (Supplemental Fig. S3). Additionally, the analysis suggests these 2 miRNAs are involved in cardiovascular system development and function, gene expression and cellular development, growth, assembly and function. Inclusion of mir-100-5p, let-7b-5p and mir-424-5p, with mir-448 and mir-199, resulted in a slight alteration of the listed pathways, but overall, the disease and functions remained consistent with the previous findings (Supplemental Fig. S4).

## Discussion

To date, this is the first rhesus macaque study to evaluate the impact of chronic in utero THC exposure on fetal brain development. In our rhesus macaque model, THC exposure did not demonstrate adverse short-term outcomes, including the absence of gross fetal anomalies, preterm birth, fetal growth restriction, or pregnancy loss. However, chronic prenatal exposure to THC was associated with subtle sex and age dependent alterations in volumetric development of the cortical plate and corpus callosum. THC-exposed fetal brains were notable for microscopic findings on histology, including increased Iba-1 and GFAP staining, suggestive of increased microglia activity, gliosis, and brain dysregulation that may have long term implications for offspring development. This is consistent with our prior reported findings, using this rhesus macaque model, that noted prenatal THC exposure alters fetal brain DNA methylation at genes involved in neurobehavioral development^20^. Also, this supports the placental-brain-axis, which suggests that many neurobehavioral disorders and neurodevelopmental disruptions are linked to pathophysiological changes in the placenta^29^. We have previously demonstrated in the rhesus macaque that THC-exposed pregnancies are associated with altered placental perfusion, fetal oxygen availability, and development^19^. In addition, our study identified two putative extracellular vesicle (EV) associated miRNAs in CSF separated by size exclusion chromatography. These two miRNAs show a greater than twofold change in fetal CSF associated with maternal THC use. These two miRNAs are involved in netrin and axonal guidance signaling, and on pathway analysis have been implicated in developmental disorders and neurological diseases. This data is indicative of subtle molecular changes consistent with the observed fetal brain histological data and implicate a potential role for miRNA regulation by THC in fetal offspring.

This is also the first study to longitudinally examine the impacts of THC on fetal brain development across gestation using in utero MRI techniques. A prior human study examined the impact of prenatal cannabis exposure on human fetal brain hippocampal functional connectivity using functional MRI at a single time point in the mid-third trimester^30^. Thomason et al.^30^, found that maternal cannabis use was associated with changes in the fetal neural connectome, specifically fetal dorsolateral, medial and superior frontal, insula, anterior temporal, and posterior cingulate connectivity. Using MRI, we assessed whether prenatal THC exposure resulted in structural brain changes across gestation and did not detect a significant main effect of prenatal THC exposure on global or regional volumetric growth of the fetal brain. It is possible that the small sample size in this study was not sufficient to detect a subtle effect resulting from THC exposure that may not manifest primarily through tissue volume changes. Alternatively, the effect of prenatal THC exposure on offspring brain development may be more pronounced during the postnatal period when maturation of the critical components of the brain that mediate cognition and executive functioning occurs^31^.

A prior human study performed non-sedated MRI on human newborns exposed to prenatal THC to examine postnatal brain maturation and infant neurodevelopmental outcomes at 12 months of age^32^. This study observed that in newborns exposed to THC in utero, brain MRI demonstrated smaller volumes in the dorsal, medial, and ventral surfaces of the frontal lobe, and dose-related increases in volumes in the lateral temporal lobe, dorsal parietal lobe, and superior frontal gyrus linked to poorer neurodevelopmental scores at 12 months of age^32^. In contrast, a prospective study using MRI neuroimaging to assess young children (ages 6–8 years old) found that prenatal cannabis exposure was not associated with global brain volume changes, including grey and white matter, but was linked to altered neurodevelopmental maturation including a thicker prefrontal cortex^33^. These neuroimaging findings correlated with altered behavioral testing in offspring prenatally exposed to THC, including cognitive deficits associated with the prefrontal cortex such as the ability to suppress response and thoughts, attention, higher-order motor control and working memory. Combined, these findings suggest that impacts on neurodevelopmental maturation from prenatal cannabis exposure may continue throughout childhood.

Our volumetric findings resembled those from other studies of fetal THC exposure in notable ways. THC-exposed male brain volumes and growth rates were, on average, lower than for females. These results were primarily from deficits in cortical plate tissue relative to control male fetuses. In contrast and to date, female THC-exposed fetuses have, on average, larger brain volumes and more rapid growth rates than controls. There is a sex bias in substance use disorders^34^ and studies in humans and mice have demonstrated sex differences in offspring following prenatal cannabis exposure, with male offspring being more affected^34,35^. Studies in adolescents exposed to THC have demonstrated subtle gender differences as well^36^. In females who used cannabis, neuroimaging was consistent with a non-significant, but slightly larger prefrontal cortex compared to control females who did not use cannabis. A larger rhesus macaque cohort is needed to better assess the degree of sex dependent sensitivity of fetal brain volume to gestational THC exposure.

Our study is also the first to link in utero imaging findings with postnatal histologic studies of prenatal THC exposure. As it is not feasible or ethical to perform in humans, much of the existing literature focuses on neuroimaging with limited correlation to histologic findings. In our study, unlike control fetuses, fetuses exposed to THC in utero demonstrated acute ischemic changes in the cerebellum, increased Iba-1 staining suggesting enhanced microglial expression, increased microglial clusters particularly in the temporal and parietal lobes, and findings suggestive of some degree of ischemic white matter injury. Microglial cells function within the neuro-inflammatory system and are vulnerable to developmental disturbances^37,38^. Our findings are consistent with prior studies of THC exposure in prior non-pregnant human and rodent studies. In non-pregnant humans, consumption of cannabinoids via inhalation has been associated with occurrence of cerebral infarcts^39–42^. In previous rodent models, THC has been demonstrated to cause intracranial vasoconstriction which may result in hypoperfusion of the brain and could be a potential mechanism for ischemic neuronal death^43^. Specifically, within a rat model, in vitro administration of THC to mitochondria isolated from rat brain led to increased oxidative stress and mitochondrial dysfunction^44^. A prior non-pregnant mouse study reported that sub-chronic THC exposure led to microglial activation that correlated with functional deficits in the cerebellar conditioned learning and motor coordination of the mice^45^. Similarly, THC treatment of non-pregnant rats induced signs of microglial activation within the prefrontal cortex with subsequent cognitive deficits observed in the adolescent rat^44^. Further pregnancy studies are needed to determine if the histologic changes of the fetal brain observed in our study correlate with long term offspring cerebrovascular health and behavioral outcomes.

Currently, a direct link between THC-induced histologic changes and long-term behavioral outcomes in humans does not exist. However, emerging human studies suggest that prenatal THC exposure is associated with offspring behavioral issues, beginning as early as the newborn period, but the underlying mechanisms are not well understood. When exposed to prenatal THC, neonates have demonstrated an increase in exaggerated startles and tremors^46^. With progression into early childhood, prenatally exposed female children have demonstrated an increased risk for aggressive behavior and attention problems^47^. Although these findings are not consistently reported across studies, some findings suggest that these behavioral differences might be subclinical in toddlers^48^. With increasing age, the reported consequences of prenatal THC on offspring are more variable. Multiple studies have demonstrated that in early childhood (ages 5–6 years old), children exposed to cannabis in utero have increased hyperactive behaviors with variable impact to their attention and memory^49,50^. The increased hyperactivity and impulsivity persists through early adolescence^51^, in addition to evidence of a predilection for psychopatholgies^52–54^.

Although the underlying etiologies for the observed in utero THC exposure-induced neurodevelopmental impacts are unknown, our study highlights a potential role for miRNAs. Prior studies have demonstrated that miRNAs play a role in early neurodevelopment including neuronal differentiation, neuronal migration, and neurite growth^55^. miRNAs are a class of small, non-protein coding RNAs that play a major role in regulating post-transcriptional gene expression^56^, and are critical for the proper development and function of organ systems and are highly expressed in the central nervous system. Multiple species of RNA, including miRNA and mRNA have been found in extracellular fluids such as CSF^57^ where they can be transported into other cells via EVs which can serve as mediators of cell-to-cell communication^58^. The interaction of miRNAs with target genes can vary depending on the target binding site, the affinity of miRNA and mRNA interactions, and the abundance of miRNAs. One limitation to the miRNA studies is that while we did separate the CSF by size exclusion chromatography using our protocol for human CSF, the amount of rhesus macaque CSF volume was not sufficient for downstream studies to evaluate the size exclusion chromatography fractions by methods such as nanoparticle tracking, vesicle flow cytometry, or transmission electron microscopy. Thus, based on this limitation, here we use the term “putative” CSF EVs in this study. In addition, we previously published that sex contributes to differences in CSF EV miRNA levels between older males and females in Alzheimer’s disease^27^. However, any potential contribution of sex to fetal CSF miRNA would require a larger set of samples, and a direct comparison between fetal and aged CSF. Thus, findings would be preliminary to speculate on in the present study. Another limitation is that it is not feasible to compute the effect sizes and confidence estimates on the small number of samples in this study (n = 5 THC, n = 3 control).

Our study’s strengths are that it utilizes a relevant translational rhesus macaque model that mimics human cannabis consumption^59,60^. Edible consumption is the second most common form of use in pregnant individuals^61^ and provides advantages of precise titration of dosing permitting blood concentrations consistent with that seen in humans while avoiding the toxins of cannabis smoke. The rhesus macaque model also limits confounding variables that are typically seen in human studies investigating THC exposure. This model allows for direct in utero assessment with MRI linked to molecular and tissue studies that would not be feasible in humans. However, the study was limited by a small cohort size due to the availability and resources needed for these non-human primate studies. Additionally, our study only focused on the prenatal period. Because the effect of in utero THC exposure on offspring brain development may become more clinically detectable in the postnatal period, the impact of prenatal THC exposure may not have been fully appreciated in this study^61,62^.

## Conclusion

As the prevalence of cannabis use in pregnancy continues to rise and safety data remains limited, there is an urgent need to further investigate the impact fetal THC exposure on fetal outcomes. This includes investigations into the molecular regulatory mechanisms of fetal response to THC exposure that may exist in other fetal tissues and systems besides the fetal CSF and central nervous system. Subtle age and sex dependent fetal brain changes were observed from prenatal THC exposure in our study, future studies leveraging our rhesus macaque model will consider incorporating functional MRI to evaluate changes in neural signaling and also assess both short-term and long-term offspring behavioral changes associated with in utero THC exposure.

## Materials and methods

### Experimental design

All protocols were approved by the Oregon National Primate Research Center (ONPRC) Institutional Animal Care and Use Committee and conformed to all guidelines for humane animal care (IP0001389). Methods are reported in accordance with the ARRIVE guidelines (https://arriveguidelines.org)^63^. The generation of pregnancies in this rhesus macaque model of THC consumption has been previously published, examining on the effects of prenatal THC exposure on placental function and development^19^, and the placental and fetal transcriptome and epigenome^20^. This study focused on the impact of in utero THC exposure on fetal neurodevelopment. As previously reported, this study used indoor-housed rhesus macaques (n = 10) maintained on a standard chow diet (TestDiet, St. Louis, Missouri)^19^. Cookies containing THC (THC edible) were made using research-grade THC obtained directly from the National Institute of Drug Abuse (NIDA)^19,64^. Tap water was available ad libitum. Edibles were administered prior to the animal’s daily chow to ensure consumption on an empty stomach and to confirm complete ingestion. Animals were slowly titrated up to 2.5 mg/7 kg/day of THC using published weight-based medical cannabis acclimation recommendations approximately 4 months prior to undergoing time-mated breeding as previously published^19,64^.

**Figure 6:**
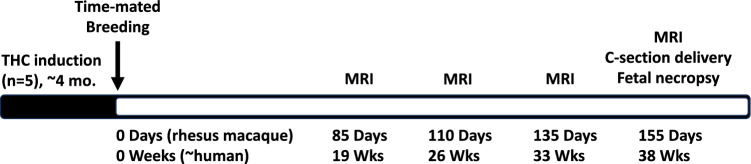
Study design overview. Timeline of the experimental design. Female rhesus macaques (n = 10, 5 THC-exposed, 5 control) underwent time-mated breeding. Fetal brain MRI was performed at gestational day 85 (G85, term is ~ 168 days), G110, G135, and G155. Immediately following imaging at G155, all animals were delivered by cesarean section delivery followed by fetal necropsy.

All animals then underwent time-mated breeding and each randomly assigned THC-exposed pregnant animal (n = 5) continued to consume a daily edible of 2.5 mg/7 kg/day throughout pregnancy. All animals (n = 10) underwent fetal brain assessment by MRI on G85, G110, G135 and G155 (Fig. 6). On gestational day 155 following imaging studies, all animals underwent immediate cesarean section delivery with fetal CSF collection and fetal necropsy for tissue collection (Fig. 6).

### Fetal brain MRI

#### MRI acquisition

In utero T2-weighted MRI was performed at G85, G110, G135 and G155 to assess fetal brain structures. All MRIs were performed approximately three hours following daily THC edible consumption. Dams were sedated with ketamine (10 mg/kg IM) and anesthesia was induced with 1.5% isoflurane vaporized in 100% O2 at a flow rate of 2 L/min. Animals were intubated and ventilated and placed head first supine into a Siemens 3T Magnetom Prisma paired with a quadrature transmit, 15-channel receive human “extremity” radio-frequency coil (QED, Cleveland, OH). Animals were kept warm with a TP500 circulating water blanket (Gaymar, Industries, Orchard Park, NY) and Bair Hugger (3M, St Paul, MN). Animal monitoring included end-tidal carbon dioxide, respiration rate, pulse rate, and O2 saturation monitoring.

T2-weighted image stacks were acquired with 0.64 mm in-plane resolution of and 1 mm slice thickness along the axial, coronal and sagittal maternal planes using half-Fourier single-shot turbo spin-echo (HASTE) sequence (Repetition time [TR] = 1200 ms, Time to Echo [TE] = 115 ms, echo train length = 74, flip angle = 150°, generalized auto-calibrating partially parallel acquisition (GRAPPA) factor = 2, and bandwidth = 650Px/Hz). Based on image quality, between 2–4 repetitions were collected per orientation.

For each fetus at each age, these multiple repetitions of motion corrupted raw T2W MR volumes (acquired in orthogonal orientations) were reconstructed to generate a single high-resolution fetal brain volume with isotropic resolution of 0.64 mm^3^ using Baby brain Toolkit (BTK)^65^. Individual reconstructed brain volumes were then registered to age and study-specific brain templates. Each template consisted of the entire sample containing control (n = 5, 3 female) and THC-exposed (n = 5, 2 female) fetuses. Actual gestational age at scan for each template was G85 (*mean [M]* = 85.9, *standard deviation [SD]* = 2.0), G110 (*M* = 110.0, *SD* = 1.6), G135 (*M* = 135.0, *SD* = 2.9), and G155 (*M* = 154.1, *SD* = 1.2). The tissue segmentation in the template space were later propagated back to individual spaces for regional volumetric analysis (Fig. 1).

#### Brain segmentation

Following slice-to-volume reconstruction, fetal brain masks were created semi-manually within ITK-SNAP^66^ by an individual not explicitly blinded to treatment group. Prior to skull-stripping, the individual brain masks were applied to the fetal head volumes for bias field correction using the N4 bias correction package^67^. For gestational age groups G85, G110, and G135, individual brain volumes were registered to the corresponding age fetal atlases^68^ available at (https://www.nitrc.org/projects/fetalmacaatlas) with rigid transformations in order to AC-PC align the brains for subsequent registrations. G155 brains were rigidly aligned to an unpublished G155 template in the G135 template space. Registrations were performed using advanced normalization tools (ANTs)^69^. Templates were constructed from the aligned brains iteratively with symmetric image normalization (SyN) diffeomorphic registrations using the ANTs buildtemplateparallel.sh script with the probability distribution similarity metric and regridded to 0.5mm^3^^70^. Anatomical segmentations from the existing fetal atlases were applied to the new study templates via linear and diffeomorphic transformations, followed by manual corrections to adapt to the new templates. Finally, the pial surface to cerebrospinal fluid, and ventral grey matter to fetal white matter boundaries were corrected with an in-house MATLAB (MathWorks Inc., Natick, MA) script inspired by Dale et al.^71^ which reclassifies voxels by intensity in a neighbor restricted manner. Specifically, it first reclassifies “island” voxels (e.g., voxels with face-based connectivity of 5 or 6 with different class voxels) to the neighboring class regardless of intensity, then reclassifies voxels with 4 neighbors of a different class to the neighbor class if the voxel intensity is greater or less than a threshold determined by the median for each tissue class.

#### Volume quantification

Fetal brain volumes were registered to the corresponding age study specific template, and the associated template segmentation label maps were applied with the corresponding linear and diffeomorphic transformations. After label application, the boundary correcting MATLAB script was implemented to address registration related interpolation errors. Volumes were calculated as the product of the number of voxels and voxel volume for a given segmented region. Total brain volume was calculated as the sum of all segmented region volumes not including external CSF (ventricular CSF volume was included). The regions segmented in this study are external CSF, cortical plate, fetal white matter, germinal matrix (GMAT), ventricles, corpus callosum, thalamus, hypothalamus, caudate, putamen, globus pallidus, hippocampus, amygdala, cerebellum, and brainstem. Growth rates were calculated as the ratio between the change in volume between subsequent scan events and the number of days between each scan event. The GMAT segmentation was exclusive to earlier gestational timepoints. Additionally, a G85 specific striatal segmentation region was created, as the caudate and putamen were not able to be individually identified until G110, with globus pallidus differentiated from the lentiform nucleus at G135. The hippocampus, amygdala, and hypothalamus were similarly not identifiable (and segmented) until G110.

### MiRNA quantification and analysis methods

#### Cerebrospinal fluid collection

Lumbar punctures were obtained from n = 5 THC and n = 3 control post-mortem fetuses performed in the lateral decubitus position with a 24-gauge Sprotte spinal needle. The CSF was collected and avoided the introduction of contaminating blood, gently mixed, and then transferred to polypropylene tubes in 0.5 mL aliquots. The CSF aliquots were flash frozen on dry ice and stored at − 80 °C until use. The CSF sample volume of 0.5 mL is not sufficient to perform immunoblots for EV markers that are routinely assessed in larger volume or pooled samples. However, we have previously performed a comprehensive analysis of human CSF to identify the optimal protocol for EV enrichment by size exclusion chromatography. Our publication shows immunoblots for size exclusion chromatography preparations using 35 nm SEC column (IZON) to fractionate 5.0 mL of CSF. Specifically, Fig. 5B validates that fractions 6–9 represent enrichment of the CSF EV pool, and that these fractions are positive for CD9, CD63, CD81, Flotillin, TSG101, and Annexin V, all protein markers for EVs.^27,28^.

#### Size exclusion chromatography

CSF was processed from n = 4 THC-exposed (one was excluded due to blood in the CSF) and n = 3 controls, as we have previously published on human CSF.^27^ CSF from 0.5 mL aliquots was thawed on ice and centrifuged for 5 min at 500× *g* in order to remove potential cellular debris. CSF aliquots were then concentrated using 30 kD cutoff ultra-filtration columns (Millipore) to a volume of 150 μL by centrifugation at 14,000× *g* for 9–11 min. Concentrated CSF samples were passed through a 35 nm size exclusion chromatography column (IZON) using 0.22 μm filtered PBS. The void volume of the column (fractions 1–5, not utilized), fractions 6–9, representing the putative CSF EV pool (800 μL), and fractions 10–25, representing the protein and liposome pool (3200 μL) were collected into sterile Lo-Bind polypropylene tubes. The fraction pools were again concentrated using 30 kD cutoff ultra-filtration columns (Millipore) to a volume of 250 μL by centrifugation at 14,000× *g* for 5 min. The protein and liposome pools were stored at − 80 °C for future analysis.

#### RNA isolation

Total RNA was isolated from 250 μL of the enriched EV pool (fractions 6–9) using the MirVana MagMax RNA Isolation Kit (Thermo Fisher) according to manufacturer instructions. Briefly, 500 μL of lysis buffer containing 0.1% beta-mercaptoethanol was added to each sample and incubated for 10 min on an orbital shaker. Magnetic RNA capture beads (30 μL) were added to the lysate and incubated for 5 min on an orbital shaker. The lysate/bead mixture was then precipitated with 480 μL of isopropanol for 20 min, samples were then placed onto a magnetic stand for 5 min, followed by two wash steps using kit wash buffers 1 and 2. Samples were treated with 50 μL of Turbo Dnase solution for 15 min. 50 μL of the re-binding buffer and 100 μL of isopropanol were added to each sample and allowed to incubate for 5 min on an orbital shaker. Samples were placed on a magnetic stand for 5 min followed one wash using kit wash buffer 1 and two washes using kit wash buffer 2. The beads were allowed to air dry for 5 min and the RNA was eluted with 20 μL of nuclease free water. Purified RNA samples were stored at – 80 °C until use.

#### Quantitative polymerase chain reaction (qPCR)

For qPCR, 3.7 μL of total RNA from each sample was converted to cDNA using the TaqMan Advanced miRNA cDNA synthesis kit protocol (Thermo Fisher). Briefly, RNA underwent a poly-adenylation step followed by a 5′ adaptor ligation. The modified RNAs were then reverse transcribed followed by a 14-cycle universal miR-amplification step. The cDNA was then diluted 1:10 with nuclease free water to a volume of 220 μL. The diluted samples were mixed with 440 μL of TaqMan Fast Advanced master mix (Thermo Fisher) and 220 μL of nuclease free water to a final volume of 880 μL. The reaction mix was then loaded onto the TaqMan Advanced MicroRNA A Card array (Thermo Fisher) containing probes for the 376 unique human miRNAs, 4 replicates of human mir-16-5p (an endogenous control candidate) and 4 non-mammalian miRNAs (optional exogenous spike-in controls included Thermo Fisher in the arrays). The qPCR amplifications and acquisition of the resulting data were performed using a QuantStudio 7 Pro Real-Time PCR instrument (Thermo Fisher).

#### miRNA expression analysis

miRNAs were analyzed using relative quantification (∆∆Cq) based on Applied Biosystems recommendations (Thermo Fisher, Part Number 4371095 Rev B). Cq values were calculated using automatic baseline and threshold values determined by ExpressionSuite Software v.1.1 (ThermoFisher). The Cq value for each well was reported along with the amplification score (AmpScore) and a Cq confidence (CqConf), which are metrics for the quality of each amplification. Prior to data analysis, amplifications were filtered based on the Cq value, AmpScore, and CqConf score for each well: (1) PCR products with a Cq > 34, or reported as ‘undetected’, were considered below the detection threshold and assigned a Cq value of ‘34’ and (2) amplifications with a Cq ≥ 34 and an AmpScore < 0.095 or a CqConf < 0.75 were excluded from analysis. Based on these criteria, miRNAs with a Cq ≤ 34, AmpScore > 0.95 and CqConf > 0.75 were deemed acceptable for further analysis. miRNAs that were not expressed in at least 80% of the samples within a group were excluded from further analysis. For miRNAs meeting all of the inclusion criteria, the following formula for calculating the ∆∆Cq for each miRNA was used: ∆∆Cq = mean ∆Cq of test samples − mean ∆Cq of CTL samples. Within each sample the ∆Cq for a miRNA was calculated by: ∆Cq = miRNA Cq − mean Cq of endogenous control miRNAs. miRNAs selected as endogenous control normalizers showed (1) stable good quality expression values in all samples regardless of experimental group and (2) best endogenous control scores in ExpressionSuite. The 2 miRNAs chosen as endogenous controls were 135a-5p and 342-3p. These 2 miRNAs were chosen using ExpressionSuite analysis software. The top 5 miRNAs that were expressed in all 7 samples were ranked based on their stability score, lower being more stable (Supplemental Table S2). For each miRNA the fold change (RQ value) was calculated by 2 − ΔΔCq. A RQ > 1 indicates increased miRNA expression in either biological group.

#### miRNA target prediction and pathway analysis

The miRDB online tool^72,73^ was used to predict targets of the miRNAs altered by exposure to THC, as this program is widely used and frequently updated (https://mirdb.org/). As pathway analysis is most effective for predictions generated from a limited gene set, predicted targets were excluded if they had a target score below 60 in miRDB. The miRDB target prediction scores range between 50–100, scores greater than 80 are considered to be most significant, while scores below 60 are to be used with caution and typically require additional evidence for inclusion. Pathway analysis was then performed on the predicted target list using Ingenuity Pathway Analysis (IPA; QIAGEN Inc., (https://www.qiagenbioinformatics.com/products/ingenuity-pathway-analysis). For IPA, we excluded cancer-related tissue and cell lines to avoid knowledge bias towards cancer in IPA. Supplemental Figs. S3 and S4 shows the IPA analysis of mir-448 and 199a-3p demonstrating associated pathways, and diseases and functions (*p* value cut off is *p* = 0.05).

### Postmortem examination and histopathology

Full necropsies were performed on all fetuses. Representative peripheral tissues, included in Supplemental Table S1, were collected and fixed in 10% neutral buffered formalin. Before removal from the cranium, the brain was flushed with normal saline followed by 4% paraformaldehyde (PFA), and then immersion-fixed in 4% PFA. Tissues were processed routinely, embedded in paraffin, sectioned at 4 µm (peripheral) or 5 µm (brain), and stained with hematoxylin and eosin for analysis by light microscopy. All peripheral fetal tissues were examined by ONPRC veterinary pathologists. Photomicroscopy was performed with a Leica DMC2900 camera and Leica LAS X microscopic imaging software. Slides were scanned using a Leica Aperio AT2 slide scanner. Histologic sections of the brain underwent routine histological examination by two neuropathologists (M.H.H. and M.R.G) blinded to exposure and outcomes.

#### Multiplex immunohistochemistry

4 µm thick sections of formalin fixed paraffin-embedded fetal macaque brains were placed onto glass slides and baked at 60 °C for 1 h and deparaffinized. Heat induced antigen retrieval using a citrate pH6 buffer (Golden Bridge International − B05C-100B) was then performed in a Biocare Decloaking Chamber at 110 °C for 15 min or in a Dako PT Module at 97 °C for 20 min. Once the decloaking chamber was cooled to 95 °C (or the PT module to 65 °C), the slides were taken out of the vessel and left to cool at room temperature for 20 min in the retrieval buffer. Slides were then washed in dH2O and TBS-T before incubation with a rabbit anti-Iba-1 antibody (Wako 019-19741) for 1 h at room temperature. Slides were then washed in TBS-T, treated with 3% H2O2 in PBS for 10 min, and incubated with the Golden Bridge International Labs Polink 1 HRP Detection System against Rabbit IgG (D13-110) for 20 min at room temperature. Iba-1 staining was visualized with the CF488 tyramide fluorophore (Biotium 92171). The attached rabbit anti-Iba-1 antibody and Polink 1 secondary antibody were stripped off the tissue section by boiling in Citrate pH6 buffer for 15 min. The slides were then incubated with a mixture of the mouse anti-NeuN (abcam ab15580), chicken anti-GFAP (Sigma AB5541), and rabbit anti-Ki67 antibodies (Abcam ab15580) overnight at room temperature. The next day the slides were washed and were incubated with secondary antibodies against mouse IgG (AF568 – Thermo Fisher A10037), chicken IgY (AF647 – Jackson ImmunoResearch 703-605-155), and rabbit IgG (Dy755 – Thermo Fisher SA5-10043) for 2 h at room temperature. The slides were then counterstained with DAPI and coverslipped with the Prolong Gold mounting media (Thermo Fisher P36930). Stained slides were imaged using a Zeiss Axioscanner at 20×. To demonstrate stripping efficiency, staining of control brain slides with only anti-Iba-1 antibody and all secondary antibodies were concurrently performed and imaged at the same exposure rate as the rest of the samples.

#### Multiplex immunohistochemistry image analysis

Image analysis was performed using HALO (Indica Labs) software. To account for both hypertrophy of individual cells as well as possible increases in cell number, the percent areas of Iba-1 and GFAP staining as proportions of the annotated prefrontal cortex and cerebellum regions were quantified as previously published,^74^ the Area quantification FL v2.3.4 module was used. For the prefrontal cortex, the frequencies of NeuN+ and Ki67+ cells in the grey matter and white matter were quantified using the Highplex FL v4.2.3 analysis module, with manual curation. To measure the percentage of Ki67+ cells, Iba-1 and GFAP staining in the cerebellar external, molecular and internal granule layers, each cerebellum was randomly sampled by dividing the tissue with a grid and regions of interest were drawn for each layer in ten randomly selected grid squares per animal. Due to the very close proximity of the granule neurons and granule cell precursors, cell segmentation by HALO was insufficiently accurate. Instead, the area of Ki67+ staining was normalized to the area of DAPI staining to represent the percentage of nuclei that are Ki67+.

### Statistical analysis

Treatment group differences in fetal birthweight and fetal tissue weights were assessed using Welch’s t-test, and in histological assessments using a Mann–Whitney t-test. All statistical tests were two-sided and used an alpha of 0.05. Linear mixed effect models (lme4 package)^75^ considering age, sex, and treatment as fixed effects including interactions, with fetus as a random effect were used to analyze regional and whole brain volumes across gestation using the R software platform (http://www.R-project.org). Reported p-values were calculated using the Satterthwaite approximation method^76^.

### Supplementary Information

Supplementary Information 1.

Supplementary Information 2.

Supplementary Information 3.

Supplementary Information 4.

Supplementary Information 5.

Supplementary Information 6.

## Data Availability

The datasets used and/or analysed during the current study available from the corresponding author on reasonable request.
