# Supplementary material for: Prenatal delta-9-tetrahydrocannabinol exposure alters fetal neurodevelopment in rhesus macaques
Source: Sci Rep. 2024 Mar 9;14:5808. doi: 10.1038/s41598-024-56386-7 (PMC10924959; doi:10.1038/s41598-024-56386-7)

Supplemental Figure 1. Multiplex immunofluorescence of the fetal prefrontal cortex. FFPE sections were stained for neurons (NeuN), microglia (Iba-1) and the proliferation marker Ki67 by multiplex immunofluorescence. **A.** 20X image showing Ki67+ cells (white arrows). **B.** 40X image.

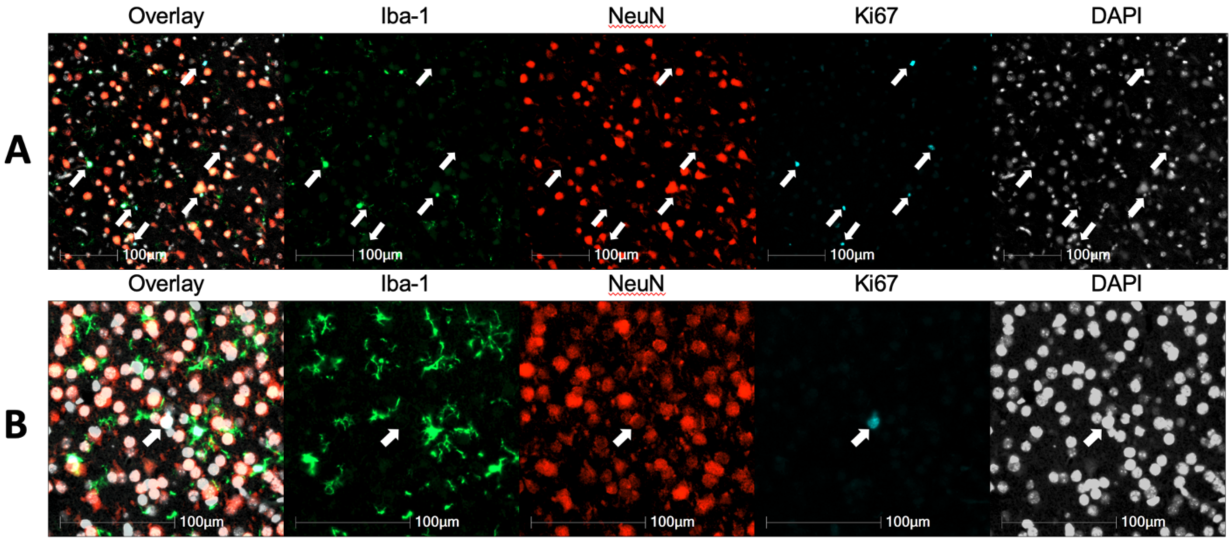

Supplement: Supplementary file 1 — Supplementary Information 1. [file 41598_2024_56386_MOESM1_ESM.pdf]
