# Supplementary material for: Prenatal delta-9-tetrahydrocannabinol exposure alters fetal neurodevelopment in rhesus macaques
Source: Sci Rep. 2024 Mar 9;14:5808. doi: 10.1038/s41598-024-56386-7 (PMC10924959; doi:10.1038/s41598-024-56386-7)

Supplemental Figure 2. Box and Whisker plots.

This study did not include an inter-assay control such as an exogeneous miRNA spike-in but previous studies utilizing CSF and/or CSF enriched EVs, demonstrated no evidence of qPCR inhibition. This study aligns with previous studies using human CSF enriched EVs, where we typically see ~60-120 miRNAs being amplified. For this data set, the average number of miRNAs for the control group was 77 and for the THC treated group the miR number was 86 and there was no significant difference between the number of amplifications that passed the qPCR performance cut-offs (p=0.7). Additionally, the mean Cq for the THC treated group was 30.6 and the control group was 31.

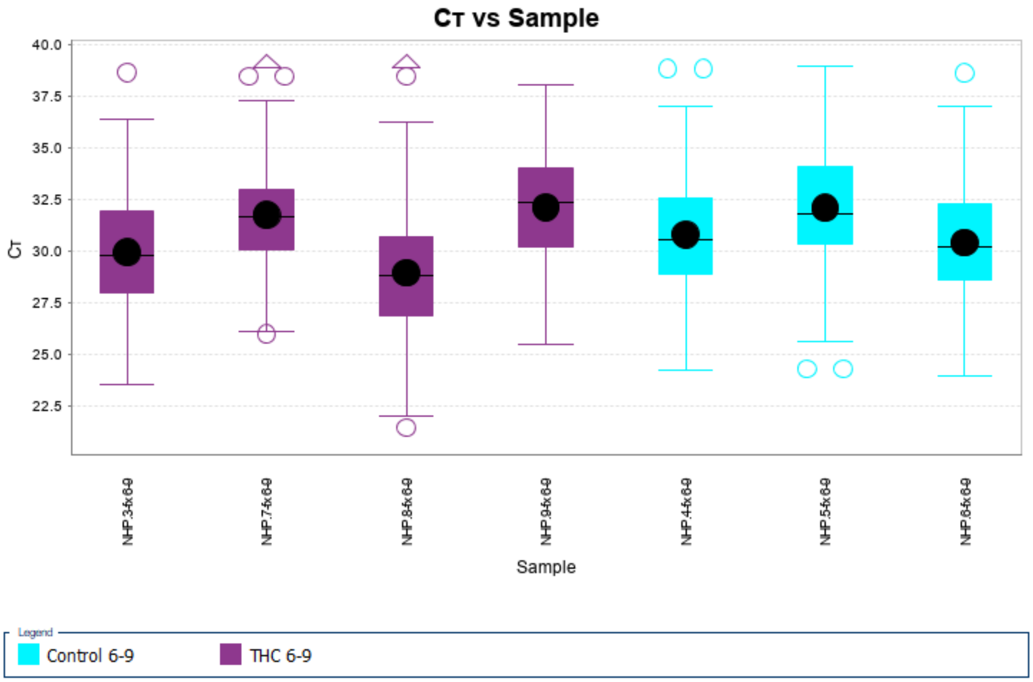

Supplement: Supplementary file 2 — Supplementary Information 2. [file 41598_2024_56386_MOESM2_ESM.pdf]
