# Supplementary material for: Prenatal delta-9-tetrahydrocannabinol exposure alters fetal neurodevelopment in rhesus macaques
Source: Sci Rep. 2024 Mar 9;14:5808. doi: 10.1038/s41598-024-56386-7 (PMC10924959; doi:10.1038/s41598-024-56386-7)
Supplement: Supplementary file 3 — Supplementary Information 3. [file 41598_2024_56386_MOESM3_ESM.pdf]

**Supplemental Figure 3.** IPA of mir-448 and 199a-3p with associated **A.** pathways and **B.** diseases and functions. P-value cut-off is set to p=0.05 (yellow line).

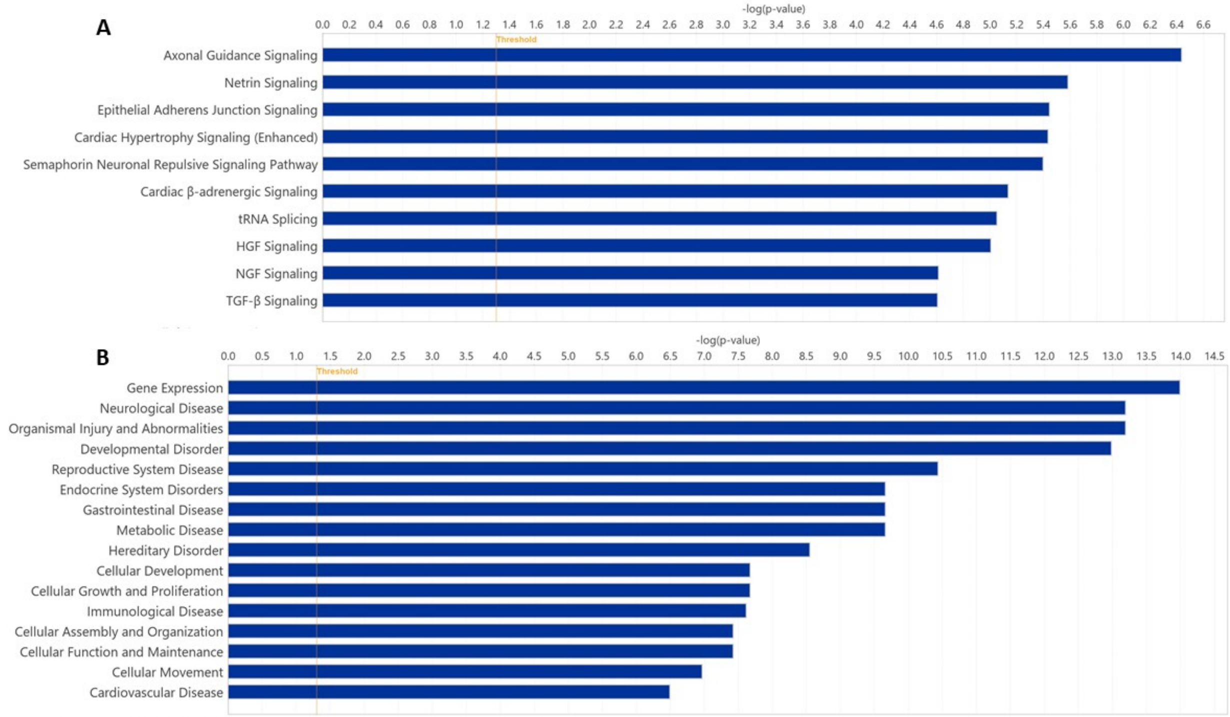


© 2000-2023 QIAGEN. All rights reserved.
