# Supplementary material for: Prenatal delta-9-tetrahydrocannabinol exposure alters fetal neurodevelopment in rhesus macaques
Source: Sci Rep. 2024 Mar 9;14:5808. doi: 10.1038/s41598-024-56386-7 (PMC10924959; doi:10.1038/s41598-024-56386-7)
Supplement: Supplementary file 4 — Supplementary Information 4. [file 41598_2024_56386_MOESM4_ESM.pdf]

Supplemental Figure 4. The figures shows the inclusion of mir-100-5p, let-7b-5p and mir-424-5p with the 2 miRNAs that were significant (mir-448 and mir-199) resulted in a slight alteration of the listed pathways but for the most part the disease and functions remained consistent with the previous findings. The addition of autophagy, senescence and HGF signaling may offer insights into other areas of organismal development disfunction and/or abnormalities.

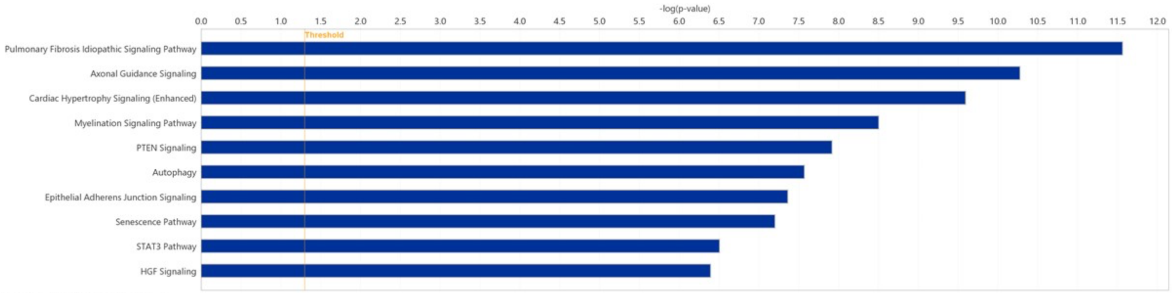

© 2000-2024 QIAGEN. All rights reserved.

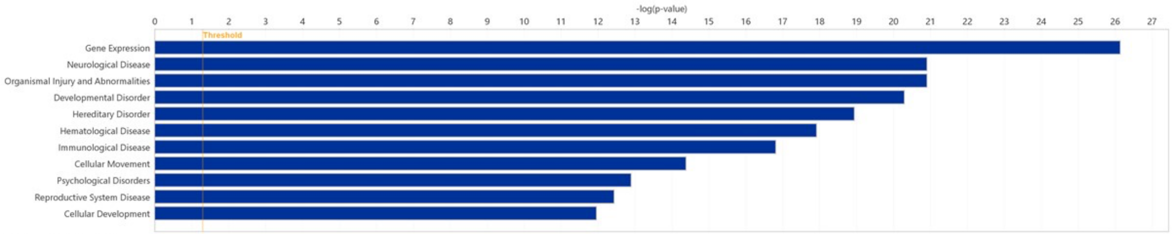

© 2000-2024 QIAGEN. All rights reserved.
