# Supplementary material for: Prenatal delta-9-tetrahydrocannabinol exposure alters fetal neurodevelopment in rhesus macaques
Source: Sci Rep. 2024 Mar 9;14:5808. doi: 10.1038/s41598-024-56386-7 (PMC10924959; doi:10.1038/s41598-024-56386-7)
Supplement: Supplementary file 6 — Supplementary Information 6. [file 41598_2024_56386_MOESM6_ESM.docx]

| **miRNA** | **miR-199a/b-3p** | | **miR-448** | | **miR-100-5p** | | **miR-424-5p** | | **let-7b-5p** | |
| --- | --- | --- | --- | --- | --- | --- | --- | --- | --- | --- |
| **BioGroup** | **Control** | **THC** | **Control** | **THC** | **Control** | **THC** | **Control** | **THC** | **Control** | **THC** |
| **Mean** | 30.01 | 28.52 | 30.41 | 31.29 | 30.6 | 28.84 | 29.44 | 30.26 | 32.7 | 30.67 |
| **Median** | 30.24 | 28.34 | 29.83 | 31.22 | 30.65 | 29 | 29.07 | 30.24 | 32.45 | 30.35 |
| **SD** | 0.8237 | 1.63 | 1.17 | 1.701 | 0.4618 | 2.839 | 0.7234 | 2.068 | 0.7375 | 1.37 |
| **Min** | 29.1 | 27.01 | 29.65 | 29.71 | 30.12 | 25.51 | 28.97 | 27.93 | 32.12 | 29.37 |
| **Max** | 30.7 | 30.37 | 31.76 | 33.03 | 31.04 | 31.86 | 30.27 | 32.63 | 33.53 | 32.6 |
| **25%** | 29.1 | 27.08 | 29.65 | 29.77 | 30.12 | 26.03 | 28.97 | 28.27 | 32.12 | 29.61 |
| **75%** | 30.7 | 30.13 | 31.76 | 32.89 | 31.04 | 31.5 | 30.27 | 32.27 | 33.53 | 32.05 |
| **N** | 3 | 4 | 3 | 4 | 3 | 4 | 3 | 4 | 3 | 4 |
| **p-value** | **0.014** | | **0.037** | | **0.054** | | **0.069** | | **0.069** | |

**Supplemental Table 2. Summary statistics of top 5 miRNAs.**
